# Impact of Information Technology–Based Interventions for Type 2 Diabetes Mellitus on Glycemic Control: A Systematic Review and Meta-Analysis

**DOI:** 10.2196/jmir.5778

**Published:** 2016-11-25

**Authors:** Nouf Sahal Alharbi, Nada Alsubki, Simon Jones, Kamlesh Khunti, Neil Munro, Simon de Lusignan

**Affiliations:** ^1^King Saud UniversityRiyadhSaudi Arabia; ^2^University of SurreyGuildfordUnited Kingdom; ^3^University of LeicesterLeicesterUnited Kingdom

**Keywords:** diabetes mellitus, medical informatics applications, technology

## Abstract

**Background:**

Information technology–based interventions are increasingly being used to manage health care. However, there is conflicting evidence regarding whether these interventions improve outcomes in people with type 2 diabetes.

**Objective:**

The objective of this study was to conduct a systematic review and meta-analysis of clinical trials, assessing the impact of information technology on changes in the levels of hemoglobin A_1c_ (HbA_1c_) and mapping the interventions with chronic care model (CCM) elements.

**Methods:**

Electronic databases PubMed and EMBASE were searched to identify relevant studies that were published up until July 2016, a method that was supplemented by identifying articles from the references of the articles already selected using the electronic search tools. The study search and selection were performed by independent reviewers. Of the 1082 articles retrieved, 32 trials (focusing on a total of 40,454 patients) were included. A random-effects model was applied to estimate the pooled results.

**Results:**

Information technology–based interventions were associated with a statistically significant reduction in HbA_1c_ levels (mean difference −0.33%, 95% CI −0.40 to −0.26, *P*<.001). Studies focusing on electronic self-management systems demonstrated the largest reduction in HbA_1c_ (0.50%), followed by those with electronic medical records (0.17%), an electronic decision support system (0.15%), and a diabetes registry (0.05%). In addition, the more CCM-incorporated the information technology–based interventions were, the more improvements there were in HbA_1c_ levels.

**Conclusions:**

Information technology strategies combined with the other elements of chronic care models are associated with improved glycemic control in people with diabetes. No clinically relevant impact was observed on low-density lipoprotein levels and blood pressure, but there was evidence that the cost of care was lower.

## Introduction

Chronic diseases such as diabetes can be managed better by implementing system-wide practices such as the chronic care model (CCM). This model identifies 6 components as essential for chronic disease management: health system organization, delivery system design, self-management support, community resources, decision support, and clinical information systems [1]. The CCM is globally applied to support system changes in diabetes management and places particular emphasis on the use of information technology [2]. Advanced information technologies enhance communication among and between health care providers and patients [3] and improve chronic disease management [4]. Various information technology applications are currently available, including electronic patient registers, electronic decision support systems, electronic medical records (EMRs), telemedicine, videoconferencing, and electronic self-management systems [5]. Advanced informatics technology can aid the monitoring of hemoglobin levels, improve clinical practices, and help eliminate the health problems caused by diabetes [6].

Several systematic reviews evaluated the potential benefits of information technology–based diabetes management interventions, and all concluded that information technology–based interventions could improve diabetes management for adult care [7-11]. However, they did not extend their focus to consider blood glucose measurements using meta-analysis techniques or map interventions incorporating CCM elements. Therefore, this systematic review aimed to determine the effect of information technology–based elements of the CCM on glycemic control in people with type 2 diabetes mellitus (T2DM).

## Methods

### Search Strategy

A comprehensive literature search was conducted using PubMed and EMBASE for articles focusing on information technology–based diabetes interventions, which were published up until July 2016. A search strategy that combined keywords and Medical Subject Headings (MeSH) using the terms “diabetes,” “diabetes mellitus,” “non-insulin-dependent,” “diabetes type 2,” and “informatics” was used. In addition, international journals were searched manually and the reference lists from retrieved articles were reviewed in order to identify additional, relevant papers (Table 1).

### Inclusion and Exclusion Criteria

Titles and abstracts of all studies identified were independently reviewed by 2 reviewers (NSA and NA) from February to July 2016. Any discrepancies between the choices of the 2 reviewers were resolved by another reviewer (SDL). The inclusion and exclusion criteria for the study are presented in the Textboxes 1 and Textboxes 2, respectively.

**Table 1:** Search strategies.

Database	Search terms	Number of studies
PubMed	1: “Diabetes Mellitus”[Mesh]	22,247
	2: “Medical Informatics Applications”[Mesh]	37,851
	1 and 2	425
EMBASE	2: 'diabetes'/exp AND 'mellitus'/exp	537,195
	1: 'information'/exp AND 'technology'/exp	28,774
	1 and 2	557

**Inclusion criteria for the study.**

- The study design specifically evaluated the use of information technology–based interventions for the management of diabetes mellitus or T2DM, but the authors also included studies where information technology was part of a comprehensive intervention in which the impact of the information technology element was reported separately
- The study focused on T2DM or both type 1 and type 2 diabetes mellitus, because T2DM accounts for more than 90% of all diabetes cases [12]
- The study reported glycated hemoglobin (hemoglobin A_1c_ or HbA_1c_) as an outcome measure
- The study had one of the following study designs: randomized controlled trial, nonrandomized controlled trial, and before-after trial

**Exclusion criteria for the study.**

- Reviews lacking original study data
- Studies that evaluated information technology–based interventions in other chronic diseases
- Studies published in languages other than English or Arabic
- Studies of children with diabetes, as very few have T2DM, or studies of pregnant women with gestational diabetes, as this is not T2DM (even though people with gestational diabetes are at an increased risk)
- Papers using the same data as those already selected for use in the review

### Data Extraction and Quality Assessment

Two reviewers (NH-NS) independently reviewed the title, the abstract, and the article. Discrepancies were resolved by consensus or determined by other reviewers (SDL). Information was taken from each study using a predesigned collection form: authors, date of the study, technology type, country, study site, duration of the intervention, type of diabetes, study design, communication type, main user, number of participants, and outcome measures. Relevant missing data were obtained from authors. A qualitative review was performed to extract information about the clinical and process outcome measures: body weight, systolic blood pressure, diastolic blood pressure, low-density lipoprotein (LDL) cholesterol, high-density lipoprotein, process of care, cost of care, patients’ satisfaction, smoking levels, and medication adherence. As part of data collection, quality assessment for each included study was conducted using PRISMA (Preferred Reporting Items for Systematic Reviews and Meta-Analyses) guidelines [13]. The studies were assigned a quality score ranging from 0 to 7 based on certain criteria (each item scored 1 point; the total score was 7), as depicted in Textbox 3.

**Criteria for assigning the quality score.**

- Whether the study design was randomized
- Whether the study described criteria for selection of participant
- Whether both groups had similar baseline
- Whether the study described the intervention methods
- Whether the study evaluated the interventions after 6 months or more
- Whether the study used intention-to-treat analysis
- Whether the study reported method of blinding

### Data Analysis

The outcome measure was the changes in HbA_1c_ levels from baseline to follow-up. HbA_1c_ is recognized as a significant indicator of information technology–based intervention effectiveness in patients with T2DM because it reflects average glycemia over 8 weeks and is strongly associated with diabetes complications [14,15]. A heterogeneity test (random-effects model) was used to evaluate variation between the studies. In addition, meta-analysis was used to assess the effectiveness of information technology–based interventions according to the type of technology used. All analyses were performed using the R Project for Statistical Computing program (AT&T Labs) [16]. HbA_1c_ is recognized as a valuable indicator of treatment effectiveness in patient with T2DM, because it reflects average glycemia over several months, unaffected by self-report bias, and strongly associated with T2DM complications [17].

## Results

### Study Selection and Characteristics

**Figure 1:**
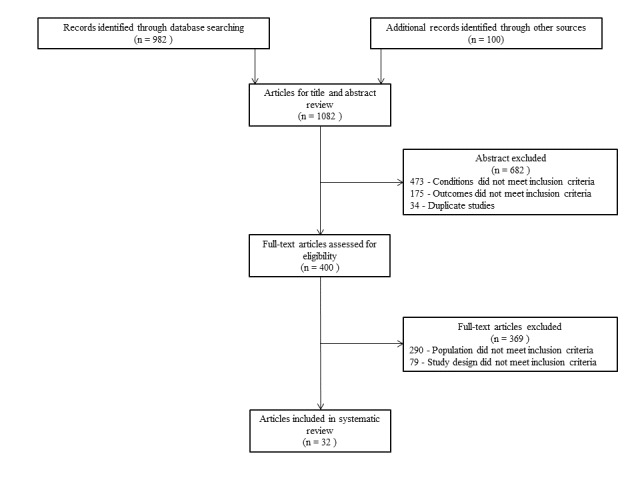
Study selection process.

The data search produced 982 studies and a further 100 studies were identified by manual searching and from the references of included articles, giving a total of 1082 studies. A flow diagram of the search and selection process is shown in Figure 1. The data search identified 1082 relevant studies, but 682 studies were excluded after title or abstract analysis. Therefore, 400 full-text studies were assessed for eligibility after excluding 34 duplicates (as well as 648 studies that did not address the topic under consideration). At the final stage of eligibility assessment, 369 articles were excluded, and the remaining 32 studies were included in this review.

All 32 studies selected for the review were published in English. Included studies had a total of 40,454 patients, more than half of them with both type 1 diabetes mellitus (T1DM) and T2DM, the others suffering from T2DM alone. Most of the included studies were conducted in the United States, while the 5 remaining studies were carried out in the United Kingdom [18], Korea [19], Germany [20], the Netherlands [21], and Canada [22], with the majority published after 2005. Study duration ranged from 3 months to 36 months; the main characteristics of the included studies are summarized in Multimedia Appendix 1. The intervention was targeted at monitoring diabetes care. As our meta-analysis was designed to specify, all studies included different types of technologies. The interventions had varying degrees of complexity. Information technology–based intervention strategies included different combinations of transmission of data, reminders, and data storage: 4 studies used a diabetes registry [18,23-25], 3 studies used EMRs [26-28], 18 articles used electronic patient self-management technology [19,29-45], and the other studies used electronic decision support systems (7 studies) [20-22,46-49].

### Applications of Technologies

Four types of technological applications were identified as constituting the information technology–based intervention: electronic self-management system, electronic decision support system, diabetes registry, and EMRs. In some studies a combination of 2 technologies was identified. However, we categorized the types based on the main technology used in such cases.

#### Electronic Self-Management System

Out of 32 articles, 18 used electronic self-management tools [19,29-45]. These studies have applied several tools designed for electronic self-management systems, and the technologies have all shown to be successful. In this category, patients made use of the Internet, mobile phones, telemedicine, or other technologies to enhance their self-management, essentially to access diabetes health education programs or to communicate with clinicians.

In this group, the best weighted mean change in HbA_1c_ level, −1.86%, was reported in the study by Smith et al [37]. To elaborate, the baseline HbA_1c_ level was 10.83% (intervention group) and 11.08% (control group; *P*<.001). HbA_1c_ level in intervention and control groups at 9 months was 7.68% and 10.83%, respectively (*P*=.02). In this study, patients used the MyCareTeam system, which gives people with diabetes the opportunity to log in and receive information about their condition, provides a portal for patients to log their blood glucose readings, and creates a space in which patients can discuss their condition with physicians and exchange information related to diabetes management. This technology was found to improve long-term glycemic control where a 1% decrease in HbA_1c_ levels is associated with a 35% decrease in nerve damage, vision loss, and kidney disease, a 22% decline in peripheral vascular disease, an 18% reduction in the likelihood of suffering a heart attack, and a 25% reduction in diabetes-related deaths of all types [37].

#### Decision Support System

Out of 32 articles, 7 used a decision support system [20-22,46-49]. Tools belonging to this system were used to process data and provide recommendations and alerts to providers and their patients. Studies in this category utilized advanced forms of technology such as telemedicine, touch screen, computer-aided assessment, and Web-based diabetes trackers. In this group 71% of studies showed improvements in glycemic levels. The best improvement in HbA_1c_ level in this group was observed in a study by Augstein et al [20] (−0.34% in the intervention group vs 0.27% in the control group; *P*<.011). This randomized trial enrolled adult patients with T1DM or T2DM and who were recruited from 5 outpatient centers. The decision support system tool that was used is the Karlsburg Diabetes Management System (KADIS). This system is an interactive, computerized, personalized decision support system for T1DM and T2DM. It allows for visualization of the current, characteristic daily HbA_1c_ profile, identification of individual weak points, and interactive simulation procedures to predict outcomes of therapeutic strategies and lifestyle changes in HbA_1c_ profiles [20].

#### Diabetes Registry

Diabetes registry was the primary intervention in 12% (4/32) of the included studies [18,23-25]. The impact of diabetes registries on improving care was difficult to quantify because the registries performed many different functions. Although several studies have demonstrated improvements in the process of care delivery, the mechanism that accounts for this improvement is far from clear. Any improvement in the HbA_1c_ level was modest [18,23-25], and strict entry criteria in another study left very little scope for improvement.

In one study, a pragmatic, cluster randomized controlled trial was conducted over a period of 15 months, with 3608 adult patients with T2DM, older than 35 years, and clients of 58 general practices from 3 localities in England. The intervention was a computerized diabetes register that incorporated the diabetes recall and management system. The registers were based on structured datasets completed on paper forms and laboratory reports. The results revealed that the intervention group demonstrated a decline in the mean level of HbA_1c_, down to 7.32%. In addition to the improvement of the clinical outcome, the study also demonstrated improvements in the clinical process, including foot examinations, 67.3% (*P*<.05); dietary advice, 46.3% (*P*<.05); and blood pressure monitoring, 71.4% (*P*<.05) [18].

Among the studies, 2 randomized controlled trials did not show a significant improvement in the levels of HbA_1c_ [24,25]. However, the first of these evaluated the effects of a registry-generated audit for diabetes, as well as feedback and patient reminder interventions on diabetes care, for 483 diabetic patients [24]. The registry was integrated electronically with other clinical information systems, automatically queried clinical databases, and reported summaries. After 12 months of evaluation, the study demonstrated that the hemoglobin levels were not different for either the intervention group or the control group.

#### Electronic Medical Record

Only 3 out of 32 studies utilized EMR as the primary technological equipment [26-28]. The EMR was used as a decision support system or was integrated with Web-based personal health records. Out of the 3 articles in this group, 2 showed improvement in clinical outcomes, with O’Connor et al highlighting the best improvements in HbA_1c_ levels. In this study the impact of EMR was evaluated over 12 months, in 11 clinics, and involving 2556 diabetic patients. The implementation of the EMR was associated with significant improvements in HbA_1c_ level (8.5%-7.9%, *P*<.011) and systolic blood pressure control but no improvement in LDL cholesterol levels [26].

### Types of Technology Used

This systematic review has identified 4 broad categories of T2DM management technologies. Electronic self-management technologies were a major component of studies targeting patients. These technologies may be placed broadly into 4 categories. The first category is the Web-based intervention that is based on interactive websites. Patients upload their data and receive feedback at a time most convenient for them and are not limited to clinic office hours [29-32,36,38,45]. The second category is the telephone-based system, where patients regularly submit data about their conditions and they receive instructions and feedback through telephone calls performed by diabetes clinicians for follow-up or drug adjustment [34,39,40]. The third category is a mobile phone–based system, where patients use their mobile phone to upload their data manually or by connected glucometer, and then all data stored can be transmitted directly to their clinicians [19,42]. The last category is the telemedicine, which is a useful technology for consulting [41].

EMRs and disease registries facilitate care providers to conduct clinical audits, provide them with reports for analyzing a patient’s key diabetes-related measures, and assist in tracking the patient’s progress. Registries are a central component of the CCM within both the public and private health sectors. Previous studies have suggested that their use correlates with improved outcomes for patients with diabetes [50]. The use of a diabetes registry can improve clinical outcomes, including HbA_1c_ levels [18,23,24]. Also, information technology has been used as a decision support system based on several tools such as clinical guidelines, condition-specific order sets, or reminders that linked to specific patient data such as blood pressure, cholesterol level, hemoglobin control, and annual eye and foot screenings, with the advice given to the physicians based on evidence-based guidelines.

### The Effects of Information Technology–Based Interventions on HbA_1c_

**Figure 2:**
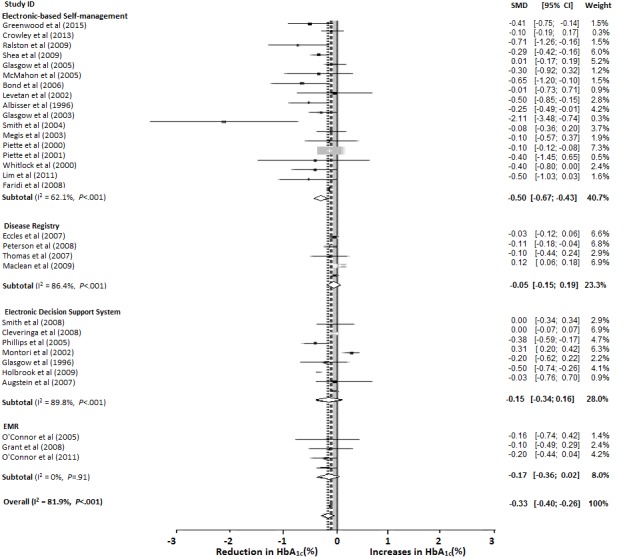
The reduction in HbA1c values by the type of information technology–based intervention. EMR: electronic medical record. Horizontal lines: confidence intervals, squares: means, diamonds: pooled estimated measures. SMD: standardized mean difference.

The overall effect of different information technology–based interventions on the mean reduction in HbA_1c_ level was 0.33% (95% CI −0.40 to −0.26, *P*<.001; Figure 2). For the 4 information technology–based interventions, studies focusing on electronic self-management systems demonstrated the largest reduction in HbA_1c_ level (0.50%), followed by those with EMRs (0.17%), an electronic decision support system (0.15%), and a diabetes registry (0.05%).

## Discussion

### Principal Findings

This study reviewed clinical trials that assessed the effect of information technology on glycemic control of patients with T2DM. This systematic review (32 studies, 40,454 patients) shows that information technologies achieved a significant reduction in glycated hemoglobin in patients with T2DM. Significant positive effects on HbA_1c_ levels were found in 30 studies. The subgroup analysis demonstrated that electronic self-management technology had the greatest impact on the health of patients with T2DM, while the diabetes registry had the least effect.

The impact of diabetes registries on improving care was difficult to quantify because the registries performed many different functions: it was unclear if the improvements had been driven by the functioning of the basic diabetes registry or other interventions. In the same way, being certain about the effectiveness of electronic health record systems is challenging because there cannot be a certain relationship with any presumed dependent variable; there is at best an association between technology use and quality and satisfaction [51]. Although some studies have demonstrated improvements in the process of care delivery, demonstrating improvements in HbA_1c_ levels has proved to be more challenging [18,23,24]. In addition, the baseline hemoglobin level in one study was 7.7% in both control and intervention groups [28]. Information technology diabetes interventions may need to be introduced to patients with a baseline HbA_1c_ level equal to or higher than 8.0% in order to effect changes, as was the case in 12 studies reported. This analysis further demonstrated a greater reduction in HbA_1c_ level in patients with a poor HbA_1c_ level as compared with a moderate one (−0.58% vs −0.20%).

These days, information technologies are advancing rapidly and are ubiquitously available worldwide. There is widespread belief that information technology may reduce care costs for patients with diabetes. However, relatively few studies have evaluated the effect of information technology on costs. The secondary outcome measures were summarized qualitatively because they were measured with various instruments. We found that a number of information technology studies reported improvements in the process of care and patient satisfaction, which suggests that information technology may be an effective strategy for changing patient behaviors. Additionally, our review demonstrates that there was no clinically relevant effect on LDL and no effect on blood pressure. This finding confirms those from a previous systematic review [7].

For diabetes care to be successfully supported by information technology–based interventions, their use should be embedded in the CCM. This review was able to map these technologies onto the CCM. It found that the most common CCM components used in trials besides the clinical information system were self-management support, delivery system design, and decision support. Health care organization and community resources were not reported. Most of the studies reported using multiple components in their interventions. It was difficult to determine which elements of the CCM benefit diabetic patients the most. However, interventions using self-management support reported the largest improvements in HbA_1c_ levels. Four components of the CCM have a stronger effect on HbA_1c_ levels than do 2 or 3 elements.

### Comparison With Prior Work

Several systematic reviews related to health information technology have been undertaken, but they have limited their scope to specific systems such as telemedicine [52], clinical decision support system [53], mobile phone [54], and EMRs [55,56]. No study to date has reviewed a broad range of health information technologies. In addition, previous systematic reviews with less methodological rigor have not performed meta-analysis or have failed to detect significant differences between different types of technological interventions [8,10]. The findings confirm the findings of meta-analyses that stated that changes must be made in multiple areas of CCM elements in order to considerably improve the quality and outcomes of diabetes care [57].

There is evidence to suggest that electronic self-management systems may improve glycemic control in patients with T2DM: this meta-analysis indicated that this type of technology significantly reduced HbA_1c_ levels compared with the control group (pooled mean difference 0.50%, *P*<.001). These results support the conclusion previously reported in 2012 [51]. It appears that clinical outcomes improve more when several CCM components are utilized simultaneously. In a review of 69 studies of diabetes care systems that used a variety of CCM components, the results demonstrated that utilizing all CCM elements may reduce the HbA_1c_ level by 0.46%, which is quite similar to our findings (−0.50%).

### Limitations

This review and meta-analysis has several advantages over most, previous systematic reviews of the impact of information technology on diabetes care. We reviewed a large body of literature, assessed the quality of included trials, and contacted authors of some studies to collect missing data. To our knowledge, this systematic review presents the first pooled analysis results of varied information technology types on HbA_1c_ levels among patients with T2DM. Nevertheless, this review also has limitations. We used HbA_1c_ level as the primary outcome measure because of its long-established association with adverse cardiovascular outcomes in diabetes [58]. However, we recognize that an appropriate process of care, as described in the CCM, may be more important in improving health outcomes. In addition, there is the possibility of publication bias as people are more likely to publish positive findings. Selection bias also consists of an exclusive focus on English- or Arabic-language studies, to the exclusion of studies in other languages. Although searches were carefully conducted using major databases and a cross-referencing method, there is the possibility that some publications were not included in the study because of the inclusion criteria. Most of the studies were conducted in the United States, with only a few conducted elsewhere. Considering that many European countries have implemented information technology interventions, it was surprising to note the lack of evaluation of these systems in diabetes care. Inevitably in this study, only HIT that was operational and part of a health system was included in our review. We know that may HIT implementations fail, and that a socio-technical approach and provide insights into why and when HIT can improve the care of patients with T2DM [59,60]. Further research needs to include how and why some implementations succeed and potentially improve health while others fail.

### Conclusions

The findings of this review suggest that, in general, information technology interventions improve glycemic control. Patient self-management support appears most promising; EMRs and clinical decision support system appear to confer benefits, but disease registries by themselves do not appear to improve quality. In addition, the results conform to presumptions surrounding the CCM that changes must be made in multiple areas in order to considerably improve the outcomes of diabetes care. However, further investigation is still required to increase our understanding of how, why, and when information technology can improve the care of patients with T2DM. This includes a cost-benefit analysis of using information technology and the other secondary outcomes.

## Multimedia Appendix 1

Summary of information technology–based interventions for type 2 diabetes.

## Abbreviations

CCM: chronic care model
EMR: electronic medical record
HbA_1c_: hemoglobin A1c
LDL: low-density lipoprotein
PRISMA: Preferred Reporting Items for Systematic Reviews and Meta-Analyses
T1DM: type 1 diabetes mellitus
T2DM: type 2 diabetes mellitus
