# Supplementary material for: Impact of Information Technology–Based Interventions for Type 2 Diabetes Mellitus on Glycemic Control: A Systematic Review and Meta-Analysis
Source: J Med Internet Res. 2016 Nov 25;18(11):e310. doi: 10.2196/jmir.5778 (PMC5148808; doi:10.2196/jmir.5778)
Supplement: Supplementary file 1 [file jmir_v18i11e310_app1.pdf]

Summary of information technology–based interventions for type 2 diabetes.

Intervention type and study	CCM[a] elements	Study design	Country	Setting	No. of patients, I/C[b]	Duration, months	Inclusion criteria	Changes in HbA_{1c} %, I vs C or pre-post	WMC	Other measures	Results	Q score
**Disease registry**												
Eccles et al [1]	CIS[c], DS[d]	Cluster RCT[e]	United Kingdom	PHC[f]	1674/1934	T2DM; older than 35 years	−0.43 vs −0.21	−0.22	−0.22	Systolic BP<br>Diastolic BP<br>LDL<br>Process of care<br>Cost of care	NS[a]<br>NS<br>NS<br>Improved<br>NS	5
Peterson et al [2]	CIS, DS, DSD[h], PSM[i]	Cluster RCT	United States	PHC	3970/3131	T1DM or T2DM; age 18-89 years	0.01 vs 0.04	−0.03	−0.03	Systolic BP<br>Diastolic BP<br>LDL<br>Process of care	Improved<br>NS<br>Improved<br>Improved	5
Thomas et al [3]	CIS, DS	RCT	United States	Clinic	252/231	T1DM or T2DM	−0.02 vs −0.01	−0.01	−0.01	Systolic BP<br>Diastolic BP<br>LDL<br>Process of care<br>Medication adherence	NS<br>NS<br>NS<br>Improved<br>Improved	5
Maclean et al [4]	CIS, DS	Cluster RCT	United States	PHC	3886/3526	T1DM or T2DM; 2 years of continuous care	0 vs −0.04	0.04	0.04	Body weight<br>Systolic BP<br>Diastolic BP<br>LDL<br>Cost of care	NS<br>NS<br>NS<br>NS<br>Improved	5
**Electronic medical record**												
O'Connor et al [5]	CIS, DS	RCT	United States	PHC	57/65	T1DM or T2DM; age 18-89 years; 3 years of continuous care	−.058 vs −0.32	−0.26	−0.26	Process of care	Improved	5
Grant et al [6]	CIS, DS	Cluster RCT	United States	PHC	126/118	People with T1DM or T2DM; HbA_{1c} > 7.0%	−0.2 vs −0.2	0	0	LDL	NS	6
O'Connor et al [7]	CIS, DS	RCT	United States	Clinic	1194/1362	Adults with T2DM	−0.6 vs −0.3	−0.3	−0.3	Systolic BP<br>Diastolic BP<br>LDL<br>Patients satisfaction	Improved<br>Improved<br>NS<br>Improved	5
**Electronic self-**												

**management system**													
	Ralston et al [8]	CIS, DS, DSD, PSM	RCT	United States	Web-based	39/35	T2DM; age 18-75 years; HbA_{1c} > 7.0%	−0.82 vs 0.2	−1.04	−1.04	Systolic BP<br>Diastolic BP<br>HDL cholesterol	NS<br>NS<br>NS	6
	Shea et al [9]	CIS, DSD, PSM	Cluster RCT	United States	PHC	844/821	T1DM or T2DM; older than 55 years	−0.31 vs −0.06	−0.25	−0.25	Systolic BP<br>Diastolic BP<br>LDL	Improved<br>Improved<br>Improved	7
	Glasgow et al [10]	CIS, DSD, PSM	Cluster RCT	United States	PHC	469/417	T2DM; older than 25 years	−0.19 vs −0.17	−0.02	−0.02	HDL	NS	6
	McMahon et al [11]	CIS, DSD, PSM	RCT	United States	MC	52/52	T1DM or T2DM; HbA_{1c} > 9.0%	−1.6 vs −1.2	−0.4	−0.4	Systolic BP<br>Diastolic BP<br>HDL cholesterol	NS<br>Improved<br>Improved	6
	Bond et al [12]	CIS, DSD, PSM	RCT	United States	Clinic	31/31	T1DM or T2DM; older than 60 years	−0.6 vs −0.06	−0.54	−0.54	Body weight<br>Systolic BP<br>Diastolic BP<br>HDL<br>LDL	Improved<br>Improved<br>Improved<br>Improved<br>Improved	4
	Levetan et al [13]	CIS, PSM	RCT	United States	Clinic	64/64	Adult with T2DM	−1.07 vs −0.6	−0.47	−0.47	Body weight<br>Systolic BP<br>Diastolic BP<br>HDL<br>LDL	NS<br>NS<br>NS<br>NS<br>NS	5
	Albisser et al [14]	CIS, PSM	NCT[j]	United States	Clinic	238/151	T1DM or T2DM	−1.1 vs 0.1	−1.2	−1.2	Body weight<br>Cost of care	NS<br>Improved	2
	Glasgow et al [15]	CIS, DSD, PSM	RCT	United States	Web-based	160/160	Adult with T2DM	−0.03 vs 0.24	−0.27	−0.27	HDL cholesterol	Improved	5
	Smith et al [16]	CIS, DSD, PSM	Pre-post[k]	United States	PHC	16/16	Adult with T1DM or T2DM	−3.15 vs −1.29	−1.86	−1.86	Body weight<br>Systolic BP<br>Diastolic BP<br>HDL<br>LDL	NS<br>NS<br>NS<br>NS<br>NS	4
	Meigs et al [17]	CIS, DS, PSM	RCT	United States	PHC	307/291	Adult with T2DM	−0.23 vs 0.15	−0.38	−0.38	Systolic BP<br>Diastolic BP<br>LDL<br>Process of care	NS<br>NS<br>Improved<br>Improved	7
	Piette et al [18]	CIS, DS, DSD, PSM	RCT	United States	PHC	124/124	Adult with T1DM or T2DM	−0.6 vs −0.3	−0.3	−0.3	Body weight<br>Process of care<br>Medication adherence	Improved<br>Improved<br>Improved	7
	Piette et al [19]	CIS, DSD, PSM	RCT	United States	PHC	146/146	Adult with T1DM or T2DM	−0.1 vs 0.1	−0.2	−0.2	Process of care<br>Patients satisfaction	Improved<br>Improved	7
	Whitlock et al [20]	CIS, PSM	RCT	United States	Clinic	15/13	Adult with T2DM;	−1.3 vs −0.9	−0.4	−0.4	Body weight	Improved	4

							HbA_{1c} > 7.0%						
	Lim et al [21]	CIS, DS, PSM	RCT	Korea	Clinic	48/49	T2DM; older than 60 years	−0.4 vs −0.1	−0.3	−0.3	Body weight<br>HDL<br>LDL<br>Patients satisfaction	NS<br>NS<br>NS<br>Improved	5
	Faridi et al [22]	CIS, DSD, PSM	RCT	United States	PHC	15/15	T2DM; older than 18 years	−0.1 vs 0.3	−0.3	−0.3	Body weight<br>Systolic BP<br>Diastolic BP	NS<br>NS<br>NS	5
	Crowley et al [23]	CIS, PSM	RCT	United States	Clinic	182/177	T2DM; older than 18 years	−0.2 vs −0.1	−0.1	−0.1	LDL<br>Systolic BP<br>Medication adherence	NS<br>NS<br>Improved	6
	Sevick et al [24]	CIS, PSM	RCT	United States	Clinic	131/132	Adult with T2DM	−0.6 vs −0.2	−0.4	−0.4	Systolic BP<br>Diastolic BP	NS<br>NS	6
	Greenwood et al [25]	CIS, DSD, PSM	RCT	United States	Web-based	45/45	Adult with T2DM; HbA_{1c} > 7.0%	−1.11 vs −0.7	−0.41	−0.41	Smoking<br>Medication adherence	NS<br>NS	6
**Electronic decision support system**													
	Smith et al [26]	CIS, DS, DSD	Cluster RCT	United States	PHC	360/279	T1DM or T2DM	−0.6 vs −0.6	0	0	Systolic BP<br>Diastolic BP<br>LDL<br>Process of care<br>Cost of care	NS<br>NS<br>NS<br>NS<br>NS	6
	Cleveringa et al [27]	CIS, DS	Cluster RCT	Netherlands	PHC	1699/1692	Adult with T2DM	−0.2 vs −0.1	−0.1	−0.1	Systolic BP<br>Diastolic BP<br>HDL<br>LDL<br>Process of care<br>Patients satisfaction	Improved<br>NS<br>NS<br>Improved<br>NS<br>NS	6
	Phillips et al [28]	CIS, DS	RCT	United States	PHC	1063/983	T2DM; HbA_{1c} > 7.0%	−0.56 vs −0.36	−0.2	−0.2	Diastolic BP<br>LDL<br>Process of care	NS<br>NS<br>Improved	6
	Montori et al [29]	CIS, DS, DSD, PSM	CCT	United States	PHC	3323/3323	T1DM or T2DM; older than 18 years	0.01 vs −0.3	0.31	0.31	Systolic BP<br>Diastolic BP<br>HDL<br>LDL<br>Process of care<br>Smoking	NS<br>NS<br>Improved<br>NS<br>Improved<br>Improved	4
	Glasgow et al [30]	CIS, DS, PSM	RCT	United States	Clinic	108/98	T1DM or T2DM; older than 40 years	−0.3 vs −0.2	−0.1	−0.1	Body weight<br>HDL<br>LDL<br>Patients satisfaction	Improved<br>Improved<br>Improved<br>Improved	5
	Holbrook et	CIS, DS	RCT	Canada	PHC	253/254	T2DM;	−0.2 vs	−0.4	−0.4	Body weight	NS	7

	al [31]						older than 18 years	0.2			Systolic BP<br>Diastolic BP<br>LDL<br>Process of care<br>Smoking<br>Patients' satisfaction	Improved<br>Improved<br>NS<br>Improved<br>NS<br>Improved	
	Augstein et al [32]	CIS, DS	RCT	Germany	PHC	24/25	T1DM or T2DM; older than 17 years	−0.34 vs 0.26	−0.6	−0.6	Body weight	NS	3

[a]CCM: Chronic Care Model.

[b]I/C: intervention/control.

[c]CIS: clinical information system.

[d]DS: decision support.

[e]RCT: randomized controlled trial.

[f]PHC: primary health care.

[g]NS: Non-Significant.

[h]DSD: delivery system design.

[i]PSM: patient self-management.

[i]NCT: nonrandomized controlled trial.

[k]Pre-post: before-after trial.

References:

1. Eccles M, Whitty P, Speed C, Steen I, Vanoli A, Hawthorne G, Grimshaw J, Wood L, McDowell D. A pragmatic cluster randomised controlled trial of a diabetes recall and management system: the DREAM Trial. Implementation Science. 2007;16(1):2-6. PMID:17306017
2. Peterson K, Radosevich D, O'Connor J, Nyman J, Prineas R, Smith S, Arneson T, Corbett V, Weinhandl J, Lange C, Hannan P. Improving diabetes care in practice: findings from the TRANSLATE trial. Diabetes Care. 2008;31(12):2238-43. PMID:18809622
3. Thomas KG1, Thomas MR, Stroebel RJ, McDonald FS, Hanson GJ, Naessens JM, Huschka TR, Kolars JC. Use of a registry-generated audit, feedback, and patient reminder intervention in an internal medicine resident clinic—a randomized trial. Journal of general internal medicine. 2007;22(12):1740-4. PMID:17973175
4. Maclean C, Gagnon M, Callas P, Littenberg B. The Vermont Diabetes Information System: a cluster randomized trial of a population based decision support system. Journal of General Internal Medicine. 2009;24(12):1303-10. PMID:19862578

5. O'Connor A, Lauren C, William A, JoAnn M, Sperl-Hillen J, Jane E. Impact of an electronic medical record on diabetes quality of care. Duncan Annals of Family Medicine. 2005;3(4):300–6. PMID:16046561
6. Grant R, Wald J, Schnipper J, Gandhi T, Poon E, Orav E, Williams D, Volk L, Middleton B. Practice-linked online personal health records for type 2 diabetes mellitus: a randomized controlled trial. Arch Intern Med. 2008; 168(16):1776-82. PMID:18779465
7. O'Connor P, Sperl-Hillen J, Rush W, Johnson P, Amundson G, Asche S, Ekstrom H, Gilmer T. Impact of electronic health record clinical decision support on diabetes care: a randomized trial. Annals of Family Medicine. 2011;9(1):12-21. PMID:21242556
8. Ralston J, Hirsch I, Hoath J, Mullen M, Cheadle A, Goldberg I. Web-based collaborative care for type 2 diabetes: a pilot randomized trial. Diabetes Care. 2009;32(2):234-9. PMID: 19017773
9. Shea S et al. A randomized trial comparing telemedicine case management with usual care in older, ethnically diverse, medically underserved patients with diabetes mellitus: 5 year results of the IDEATel study. Journal of the American Medical Informatics Association. 2009;16(4):446-56. PMID:19390093
10. Glasgow R, Wagner E, Schaefer J, Mahoney L, Reid R, Greene S. Development and validation of the Patient Assessment of Chronic Illness Care (PACIC). Medical Care. 2005;43(5):436-44. PMID:15838407
11. McMahon G, Gomes H, Hickson Hohne S, Levine B, Conlin P. Web-based care management in patients with poorly controlled diabetes. Diabetes Care. 2005;28(7):1624-9. PMID:15983311
12. Bond G, Burr R, Wolf F, Price M, McCurry SM, Teri L. The effects of a web-based intervention on the physical outcomes associated with diabetes among adults age 60 and older: a randomized trial. Diabetes Technol. 2007;9(1):52–59. PMID:17316098
13. Levetan C, Dawn K, Robbins D, Ratner R. Impact of computer-generated personalized goals on HbA(1c). Diabetes Care. 2002;25(1):2–8. PMID:11772893
14. Albisser A, Harris R, Sakkal S, Parson I, Chao S. Diabetes intervention in the information age. Med Inform (London). 1996;12(4):297–316. PMID:9179834
15. Glasgow R, Boles S, McKay H, Feil E, Barrera M. The D-Net diabetes self-management program: long-term implementation, outcomes, and generalization results. Preventive Medicine. 2003;36(4):410-19. PMID:12649049
16. Smith K, Levine B, Clement S, Hu, MJ, Alaoui A, Mun S. Impact of Mycareteam for poorly controlled diabetes mellitus. Diabetes Technology & Therapeutics. 2004;6(6):828-35. PMID:15684636
17. Meigs J, Cagliero E, Dubey A, Murphy-Sheehy P, Gildesgame C, Chueh H. A controlled trial of web-based diabetes disease management: the MGH diabetes primary care improvement project. Diabetes Care. 2003:26(3):750-7. PMID:12610033
18. Piette J, Weinberger M, McPhee S, Mah C, Kraemer F, Crapo L. Do automated calls with nurse follow-up improve self-care and glycemic control among vulnerable patients with diabetes? Am J Med. 2000;108(1):20–7. PMID:11059437
19. Piette J, Weinberger M, Kraemer F, McPhee S. Impact of automated calls with nurse follow-up on diabetes treatment outcomes in a Department of Veterans Affairs Health Care System: a randomized controlled trial. Diabetes Care. 2001;24(2):202-8. PMID:11213866
20. Whitlock W, Brown A, Moore K, Pavliscsak H, Dingbaum A, Lacefield D, Buker K, Xenakis S. Telemedicine improved diabetic management. Military Medicine. 2000;165(8):579-84. PMID:10957848
21. Lim S, Kang S, Shin H, Lee H, Won Yoon J, Yu S, Kim S, Yoo S, Jung H, Park K, Ryu J, Jang H. Improved glycemic control without hypoglycemia in elderly diabetic patients using the ubiquitous healthcare service, a new medical information system. Diabetes Care. 2011;34(2):308-13. PMID:21270188
22. Faridi Z, Liberti L, Shuval K, Northrup V, Ali A, Katz L. Evaluating the impact of mobile telephone technology on type 2 diabetic patients' self-management: the NICHE pilot study. Journal of Evaluation in Clinical Practice. 2008;14(3):465-9. PMID:18373577

23. Crowley J et al. The Cholesterol, Hypertension, And Glucose Education (CHANGE) study: results from a randomized controlled trial in African Americans with diabetes. American heart journal. 2013;166(1):179-86. PMID:23816038
24. Sevick A et al. Biophysiologic outcomes of the enhancing adherence in type 2 diabetes (ENHANCE) trial. Journal of the Academy of Nutrition and Dietetics. 2012;112(8):1147-57. PMID:22818724
25. Greenwood DA1, Blozis SA, Young HM, Nesbitt TS, Quinn CC. Overcoming clinical inertia: a randomized clinical trial of a telehealth remote monitoring intervention using paired glucose testing in adults with type 2 diabetes. Journal of medical Internet research. 2015;21;17(7):e178. PMID:26199142
26. Smith S, Shah N, Bryant S, Christianson T, Bjornsen S, Giesler P, Krause K, Erwin P, Montori VM. evidens research group. Chronic Care Model and shared care in diabetes: randomized trial of an electronic decision support system. Mayo Clinic Proceedings. 2008;83(7):747-57. PMID:18613991
27. Cleveringa F, Gorter K, van den Donk M, Rutten E. Combined task delegation, computerized decision support, and feedback improve cardiovascular risk for type 2 diabetic patients: a cluster randomized trial in primary care. Diabetes Care. 2008;31(12):2273-5. PMID:18796619
28. Phillips L, Ziemer D, Doyle J, Barnes C, Kolm P, Branch W, Caudle J, Cook C, Dunbar V, El-Kebbi I, Gallina D, Hayes R, Miller C, Rhee M, Thompson D, Watkins C. An endocrinologist-supported intervention aimed at providers improves diabetes management in a primary care site: improving primary care of African Americans with diabetes (IPCAAD) 7. Diabetes Care. 2005: 28(10):2352-60. PMID:16186262
29. Montori V, Dinneen S, Gorman C, Zimmerman B, Rizza R, Bjornsen S, Green E, Bryant S, Smith S. Translation Project Investigator Group. The impact of planned care and a diabetes electronic management system on community-based diabetes care: the Mayo Health System Diabetes Translation Project. Diabetes Care. 2002; 25(11):1952-7. PMID:12401738
30. Glasgow R, Toobert D, Hampson E. Effects of a brief office-based intervention to facilitate diabetes dietary self-management. Diabetes Care. 1996;19(8): 835-42. PMID: 8842601
31. Holbrook A, Lehana T, Karim K, Dolovich L, Bernstein B, Chan D, Troyan S, Foster G, Gerstein H. Individualized electronic decision support and reminders to improve diabetes care in the community: COMPETE II randomized trial. Canadian Medical Association Journal. 2009;7;181(1-2):37–44. PMID:19581618
32. Augstein P, Vogt L, Kohnert D, Freyse, E, Heinke P, Salzsieder E. Outpatient assessment of Karlsburg diabetes management system-based decision support. Diabetes Care. 2007;30(7):1704-8. PMID:17468357
